# Upconversion Luminescence Properties of Pr^3+^-Doped BaYF_5_ Nanoparticles Prepared by Microwave Hydrothermal Method

**DOI:** 10.1021/acs.inorgchem.3c03821

**Published:** 2024-01-31

**Authors:** Nadiia Rebrova, Patrycja Zdeb, Karol Lemański, Bogusław Macalik, Oleksii Bezkrovnyi, Przemysław J. Dereń

**Affiliations:** Institute of Low Temperature and Structure Research, Polish Academy of Science, ul. Okólna 2, 50-422 Wrocław, Poland

## Abstract

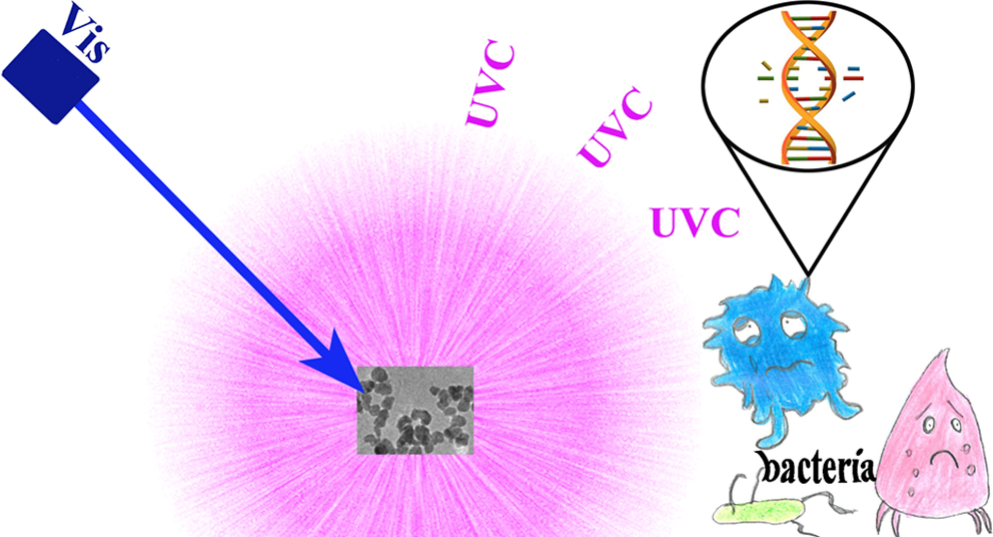

Pure and Pr^3+^-doped BaYF_5_ nanoparticles were synthesized by the microwave hydrothermal method. The nanoparticles were characterized by X-ray diffraction (XRD), transmission electron microscopy (TEM), and optical spectroscopy. The XRD and TEM confirm that the average size of nanoparticles is in the range of 26–37 nm. The optical excitation and luminescence spectra of BaYF_5_:Pr^3+^ nanoparticles are presented in the visible and ultraviolet (UV) range. It has been verified that Pr^3+^ ions are capable of emitting UV-C photons when excited by a 444 nm laser. This emission arises from a two-photon energy-transfer upconversion mechanism. The concentration dependence of the upconversion luminescence of BaYF_5_:Pr^3+^ was studied.

## Abstract 2

BaYF_5_:Pr^3+^ nanoparticles, with an average size of 26–37 nm, were successfully synthesized via the microwave hydrothermal method and their luminescent properties were studied in the UV–vis range. Despite their small size, these nanoparticles displayed remarkable UVC emission when exposed to either UV or blue-light excitations. We have discovered the effective upconversion of 444 nm excitation into UVC, which holds significant potential in various technological and medical applications.

## Introduction

Ultraviolet (UV) radiation is an important area of research as it has found many implementations in basic research in physics and chemistry, and applications in industry, notably in the production of microprocessors, and finally in medicine due to its disinfecting properties. Depending on the type and genus of microorganisms, almost all viruses and bacteria are destroyed by UV emission. Radiation in the UV-C range (100–280 nm) is particularly effective in this regard. It is much more destructive and mutagenic than UV-A (315–400 nm) and UV-B (280–315 nm) as it has high energy above 4.4 eV and damages DNA with the formation of photoproducts. These photoproducts can interfere with DNA replication and transcription, potentially leading to mutations and apoptosis. UV-C radiation can be generated by using phosphors excited by X-rays. Such nanoparticles could be used in medicine for the treatment of tumors resistant to radiotherapy. Therefore, recently, the interest of researchers in the development and production of nanomaterials exhibiting UV-C luminescence has been growing. Among the rare-earth ions, the praseodymium ion (III) is commonly used as an activator in UV-C phosphors due to its strong luminescence in the region of 220–285 nm.

A powerful feature of the rare-earth elements is that their 4f energy levels are almost independent of the host. Since the phonon energy of halides—fluorides (<600 cm^–1^), chlorides (<300 cm^–1^), bromides (<190 cm^–1^), and iodides (<160 cm^–1^)—is significantly lower compared to oxygen-containing matrices, they are good hosts for highly efficient luminescence due to the low probability of nonradiative phonon-assisted transitions.^1,2^ Among all halides, fluorides have the highest thermal and chemical stabilities and radiation resistance. All yttrium-based fluorides are of considerable interest for the Y^3+^ ion has a similar ionic size and the same charge as the lanthanide ions. Lanthanide ions can occupy the same sites in the crystal lattice without changing the crystal structure.

Compounds and solid solutions of the BaF_2_–YF_3_ binary system are excellent hosts for optically active Pr^3+^ ions due to the large band gap.^3,4^ The system is characterized by the existence of two compounds, BaY_2_F_8_ (monoclinic lattice, SG: *C*_2_/*m*) and Ba_4_Y_3_F_17_ (trigonal lattice, SG: *R*3̅), and a fluorite solid solution of Ba_1–*x*_Y_*x*_F_2+*x*_, *x* = 0.35–0.75 (cubic lattice, SG: *Fm*3̅*m*).^5−7^ So far, the luminescent and thermoluminescent properties of Pr^3+^ have been reported only in BaY_2_F_8_ and Ba_4_Y_3_F_17_ matrices, respectively.^8,9^ The Pr-doped BaY_2_F_8_ crystal was also investigated as a diode-pumped orange-emitting laser.^10^

There are several articles in the literature devoted to the optical properties of BaYF_5_ doped with Yb^3+^, Ho^3+^, Er^3+^, and Tm^3+^.^11−13^ Although the photoluminescent and radioluminescent properties of transparent glass ceramics containing BaYF_5_:Pr^3+^ nanocrystals have been studied,^14^ Karbowiak and Cichos^15^ and Fedorov et al.^16^ question the existence of BaYF_5_. Indeed, the BaF_2_–YF_3_ phase diagram does not feature the BaYF_5_ compound, and one can expect the formation of a Ba_0.5_Y_0.5_F_2.5_ solid solution.^17,18^ Since BaYF_5_ is more widely known in the literature, we decided to use this formula instead of Ba_0.5_Y_0.5_F_2.5_.

While there have been studies on BaYF_5_:Pr^3+^, our research provides a fresh perspective. Most notably, it reveals novel findings concerning the visible-to-UVC upconversion luminescence of Pr^3+^ in BaYF_5_ under 444 nm laser excitation. This phenomenon, as well as the optical characteristics in the UV range, has not been previously explored and holds potential implications in various fields, including UV-sensitive applications in medicine and industry. In this work, nanophosphors BaYF_5_ with different concentrations of Pr^3+^ were synthesized by the microwave hydrothermal method. The structure, morphology, and luminescence properties are studied.

## Experimental Details

### Sample Preparation

**Figure 1:**
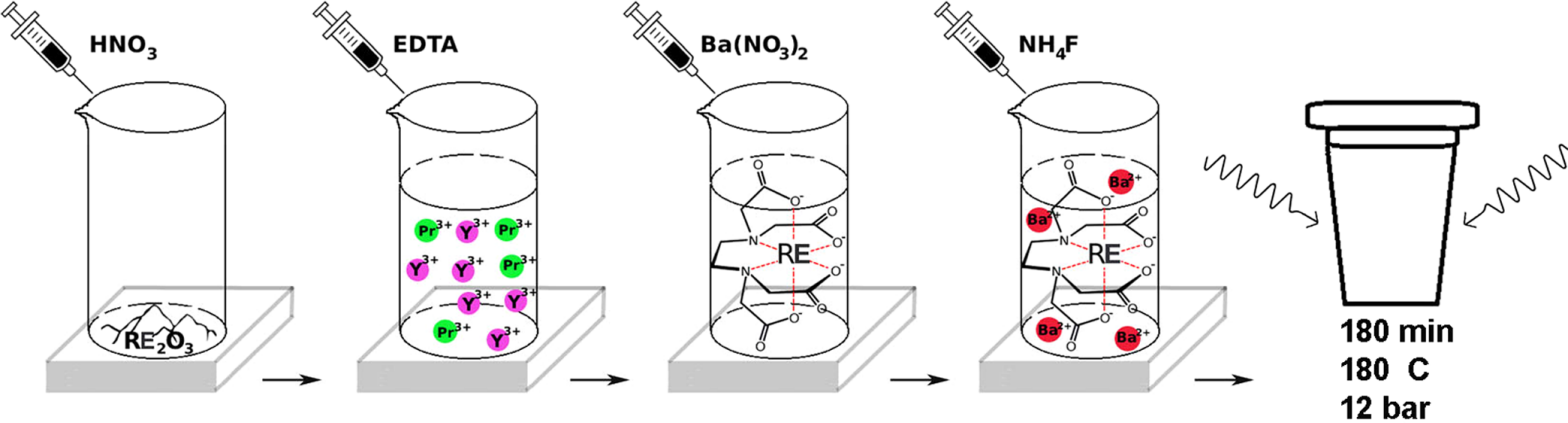
Schematic illustration of the formation of BaYF_5_:xPr^3+^ particles.

BaYF_5_:*x*Pr^3+^ (*x* = 0, 0.001, 0.005, 0.01, 0.015, and 0.02) nanoparticles were synthesized in an ERTEC Magnum II microwave reactor (frequency, 2.45 GHz; power, 600 W). Ba(NO_3_)_2_, Y_2_O_3_, Pr_2_O_3_, NH_4_F, and C_10_H_14_N_2_Na_2_O_8_ (EDTA) were used as raw materials. First, RE(NO_3_)_3_ precursors were prepared by dissolving a stoichiometric amount of oxides in nitric acid. After dissolving, a certain amount of EDTA (in a ratio 1:1 to RE) was added to the solution as a surfactant. To prevent dissociation of RE–EDTA complexes and subsequent rapid precipitation of fluorides, the pH of the solution was adjusted to 6 by adding a solution of NH_4_OH (2M). A stoichiometric amount of Ba(NO_3_)_2_ was added to this mixture. Finally, NH_4_F (1M) was added dropwise to this solution, with a stoichiometric excess of 20%. The resulting transparent solution was transferred and kept in the microwave reactor for 3 h at 180 °C and 12 bar. After completion of the reaction, white precipitates were separated by centrifugation at 10,000 rpm for 15 min, washed three times with deionized water, and dried at 50 °C in an oven for 12 h. No unusual hazards were noted during the synthesis of the nanoparticles. Figure 1 schematically shows the stages of the preparation of BaYF_5_:*x*Pr^3+^ nanoparticles.

### Characterization

Structural characterization was performed using an X’Pert PRO X-ray diffractometer with monochromatic Cu Kα radiation (λ = 1.54056 Å) and a 2θ scan range from 5 to 90°. The crystallite size of nanoparticles was estimated from X-ray diffraction (XRD) data by the Scherrer equation and the Williamson–Hall (W–H) method using the following relation^19,20^
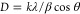
1
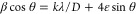
2where *D* is the particle size, *k* is the shape factor (0.9 for a spherical shape), λ is the X-ray wavelength (λ = 1.54056 Å), β is the full width at half-maximum of the most intense diffraction peaks, θ is the Bragg diffraction angle of the corresponding peaks, and ε is the strain.

The morphology of the samples was determined by transmission electron microscopy (TEM), using a Philips CM-20 SuperTwin instrument operating at 160 kV. Specimens were prepared by dispersing the sample in methanol and placing a droplet of the suspension on a copper microscopy grid covered with carbon. The samples were then dried and purified in oxygen/hydrogen plasma in a plasma cleaner. Diffuse reflectance spectra were measured using an Agilent Cary 5000 UV–visible-near-infrared (UV–vis-NIR) spectrophotometer equipped with a Harrick Praying Mantis diffuse reflection attachment. Al_2_O_3_ powder with an average grain size of 500 nm was used as the reference. Spectral resolutions of 1 and 0.5 nm were used for IR and UV–vis ranges, respectively. The inductively coupled plasma (ICP) measurement was conducted using a Thermo Scientific iCAP 7000 series spectrometer equipped with ICP-optical emission spectrometry (ICP-OES) technology. Measurements of the excitation and emission spectra in the vis range were carried out on an FLS1000 fluorescence spectrometer by using a 450 W ozone-free xenon lamp as an excitation source. The same spectrometer was used to record the decay kinetics; a xenon flash lamp was used as the excitation source. The VUV spectroscopic data were recorded on a McPherson spectrometer with a 150 W D_2_ lamp. The upconversion emission was collected using a McPherson spectrometer, and the excitation was performed with a 444 nm laser, focused on an area of 0.75 mm^2^.

## Results and Discussion

**Figure 2:**
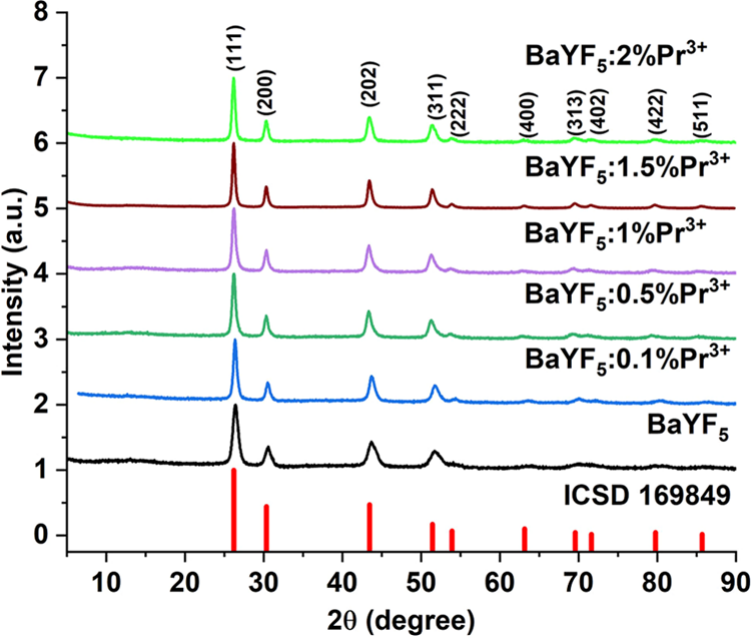
X-ray diffraction patterns of the BaYF_5_:Pr^3+^ particles and the standard reference BaYF_5_.

Figure 2 presents the results of X-ray diffraction studies of BaYF_5_:Pr^3+^ particles. The position of all diffraction peaks corresponds to the standard structure data JCPD 169849.^21^ The crystal structure of the phase is cubic (SG: *Fm*3̅*m*, no. 225), and no peaks corresponding to any other material are observed. BaYF_5_ has a face-centered cubic lattice, Ba^2+^ and Y^3+^ ions occupy half of the same Wyckoff 4a site.^21^ Lattice parameters and crystal size estimated by the Scherrer equation and the W–H method are summarized in Table 1. With an increase in the concentration of the activator in nanoparticles, an increase in the lattice parameters becomes obvious, since the radius of Pr^3+^ ions (1.126 Å) is larger than the radius of Y^3+^ ions (1.019 Å).^22^

**Table 1:** Lattice Parameters, Determined Praseodymium(III) Content by ICP-OES, Calculated Crystallite Size, and TEM Particle Size of BaYF_5_:Pr^3+^ Particles

			crystallite size (nm)			
sample	Pr^3+^ content by ICP-OES (mol %)	*a* (Å)	from Scherrer formula	from W–H plot	ε (no unit) ×10^–3^	particle size (nm) from TEM
BaYF_5_		5.8334	9.7	27.3	9.9	26.8
BaYF_5_:0.1%Pr^3+^	0.096 ± 0.005	5.8501	13.7	28.6	6.0	28
BaYF_5_:0.5%Pr^3+^	0.51 ± 0.02	5.8530	14.4	34.3	6.2	33.5
BaYF_5_:1%Pr^3+^	1.00 ± 0.05	5.9158	14.7	36.7	6.3	34.9
BaYF_5_:1.5%Pr^3+^	1.46 ± 0.07	5.9488	15.1	37.0	6.4	36.5
BaYF_5_:2%Pr^3+^	1.88 ± 0.09	5.9670	15.4	37.4	6.5	37

The difference between Y^3+^ and Pr^3+^ ions also explains the slight increase in the size of nanoparticles with an increasing praseodymium(III) content. It should be noted that the particle size estimated by the W–H method is almost twice as large compared to the particle size estimated by the Scherrer method. This can be explained by the fact that the W–H method considers lattice strain and lattice defects. The praseodymium(III) content in the samples of BaYF_5_:Pr^3+^ was determined by ICP-OES (Table 1). The experimentally obtained praseodymium content in the samples agrees well with the calculated content.

**Figure 3:**
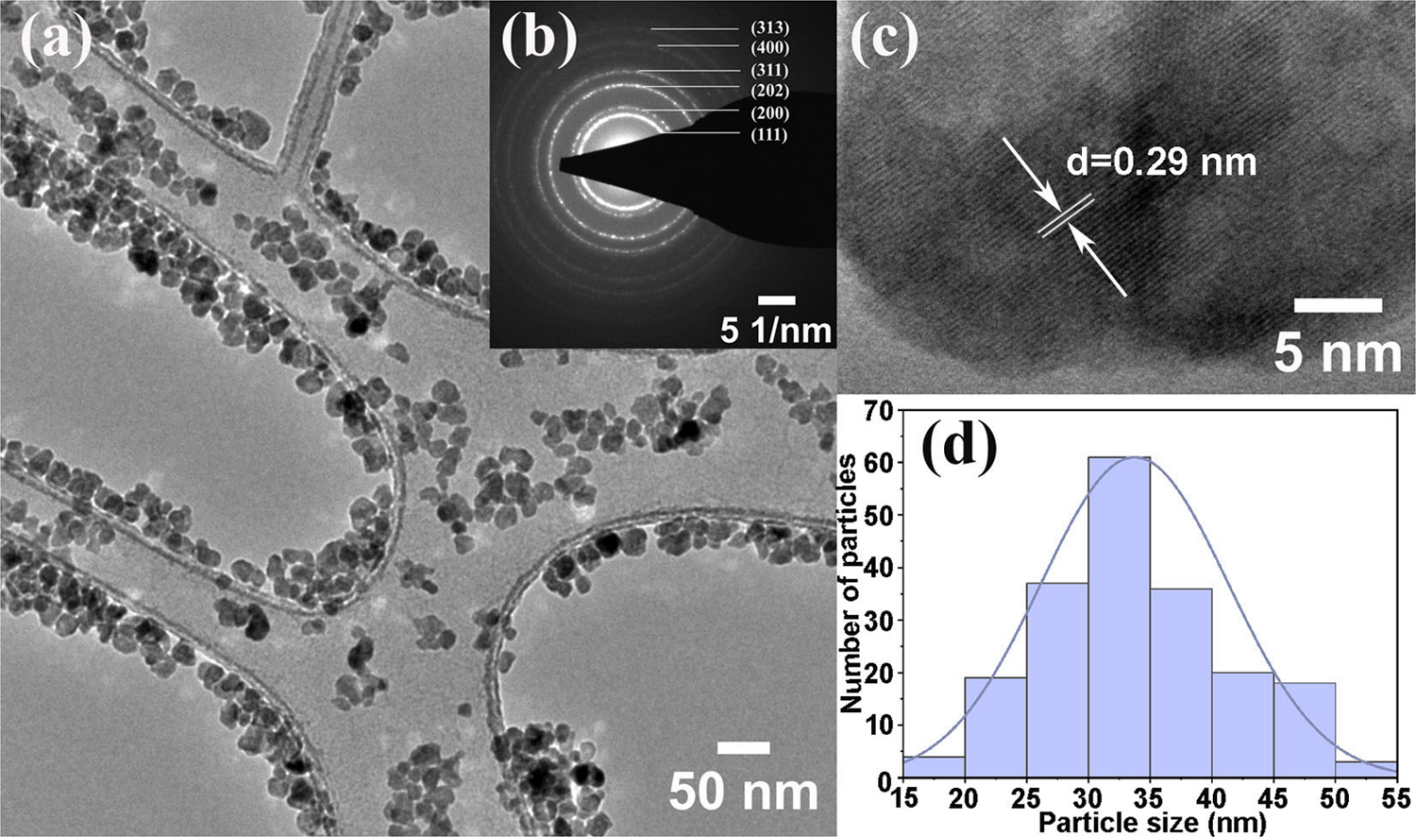
(a) TEM image, (b) SAED pattern, (c) high-resolution TEM image, and (d) particle-size distribution histogram of BaYF_5_:1%Pr^3+^.

Figure 3a displays the TEM image pattern of the BaYF_5_:1%Pr^3+^ samples. The micrograph shows that the nanoparticles have an approximately spherical shape with smooth surfaces. Particle-size distribution was evaluated by ImageJ processing (Figure 3d). The mean size is about 35 nm, which is in close agreement with the result obtained from powder XRD data using the W–H method (Table 1). The selected area electron diffraction (SAED) shows a ring pattern with diffraction spots on the rings (Figure 3b). The presence of rings and spots confirms that the nanoparticles have a crystalline nature and a well-defined structure with a good orientation within the crystallites. The SAED pattern revealed that the diffraction rings of the BaYF_5_:1%Pr^3+^ sample exhibited Debye–Scherrer rings assigned to (111), (200), (202), (311), (400), (313), respectively. The high-resolution TEM image of BaYF_5_:1%Pr^3+^ clearly shows parallel lattice fringes, which confirm the single crystallinity of the nanoparticles (Figure 3c). The distance between the atomic planes (∼0.29 nm) corresponds to the (200) planes of the cubic structure.

**Figure 4:**
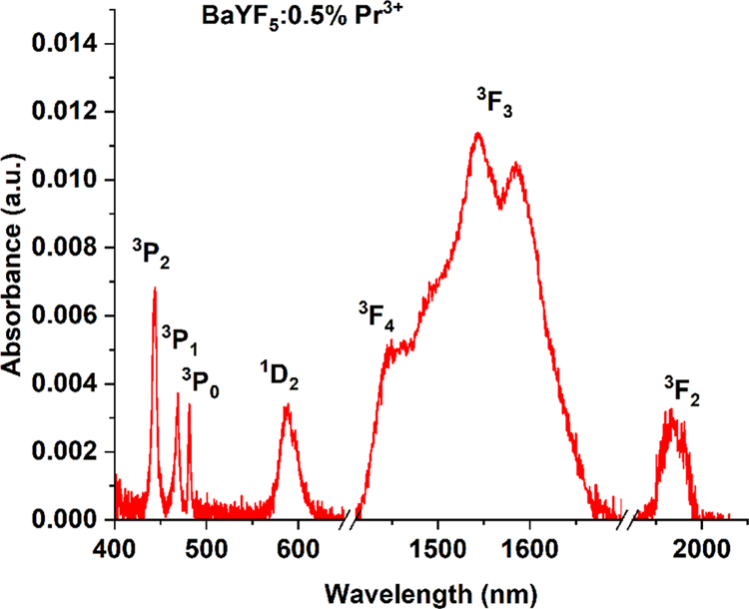
300 K absorption spectrum of the BaYF_5_:0.5%Pr^3+^ sample is derived from a reflection spectrum.

Figure 4 shows the absorption spectrum of the BaYF_5_:0.5%Pr^3+^ sample derived from a reflection spectrum recorded at room temperature in the spectral range of 400 to 2000 nm. The spectrum contains seven absorption bands originating from the ground state (^3^H_4_) to excited levels, namely, ^3^H_4_ → ^3^P_2_ (444 nm/22,522 cm^–1^), ^3^H_4_ → ^3^P_1_ (469 nm/21,322 cm^–1^), ^3^H_4_ → ^3^P_0_ (481 nm/20,790 cm^–1^), ^3^H_4_ → ^1^D_2_ (588 nm/17,006 cm^–1^), H_4_ → ^3^F_4_ (1445 nm/6920.4 cm^–1^), ^3^H_4_ → ^3^F_3_ (1497 nm/6680 cm^–1^, 1540 nm/6493 cm^–1^, 1582 nm/6321 cm^–1^), and ^3^H_4_ → ^3^F_2_ (1969 nm/5078 cm^–1^).

**Figure 5:**
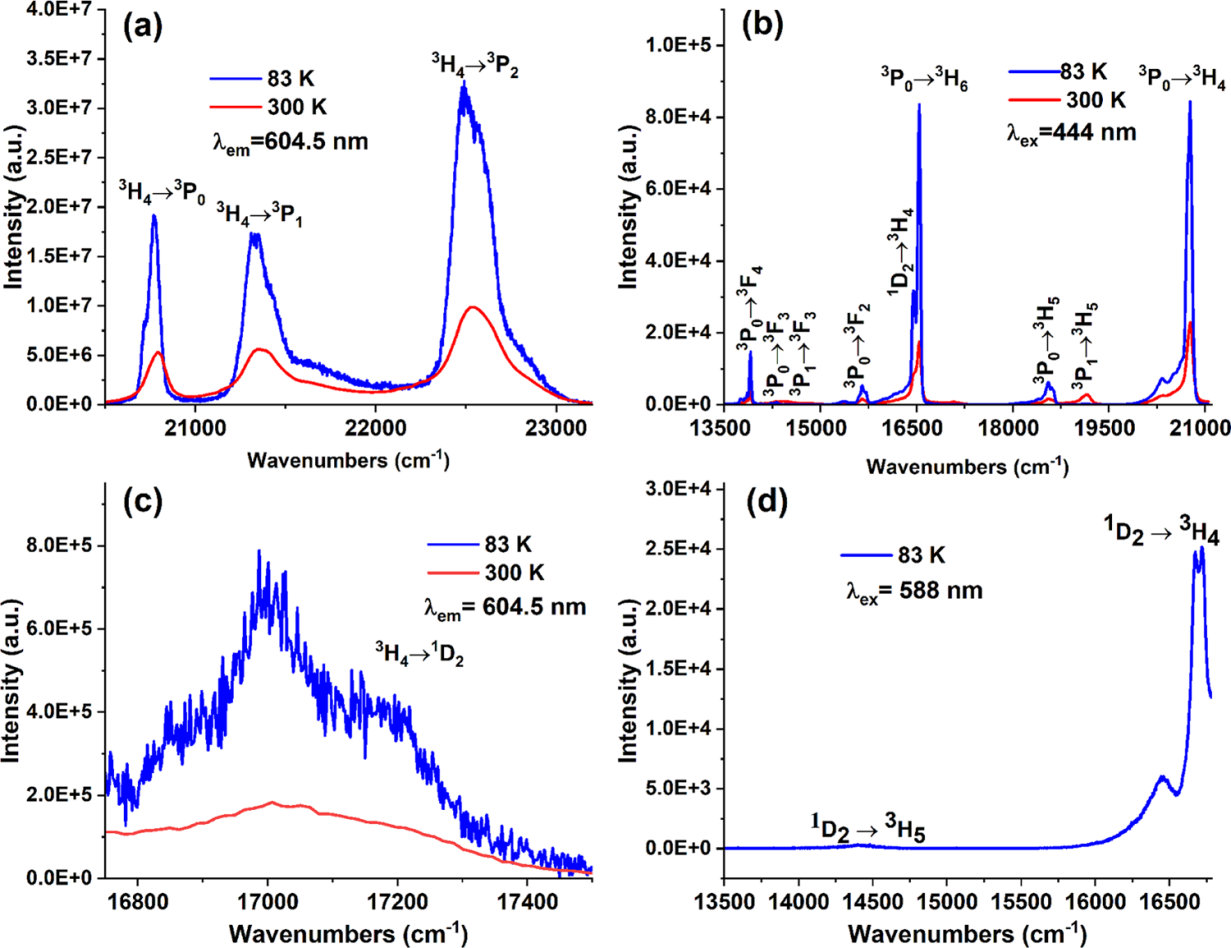
Photoluminescence (a) excitation and (b) emission spectra of BaYF_5_ doped with 1%Pr^3+^ at 83 K and room temperature. (c) Excitation spectra (λ_em_ = 604.5 nm) of BaYF_5_:1%Pr^3+^ nanoparticles at room temperature and 83 K. (d) Emission spectrum measured at 83 K under selective excitation at the ^1^D_2_ level (λ_ex_ = 588 nm).

Under monitoring the wavelength of 604.5 nm at 83 and 300 K, the excitation spectrum of BaYF_5_ doped with 1%Pr^3+^ is presented in Figure 5a. The spectrum consists of three characteristic Pr^3+^ excitation bands associated with the ^3^H_4_ → ^3^P*_j_* (*j* = 0, 1, 2) transition. The excitation bands at 83 K become narrower and better resolved compared to those at room temperature. Figure 5b shows the emission spectra of BaYF_5_:1%Pr^3+^ nanoparticles in the 460–750 nm (21739–13333 cm^–1^) spectral region upon 444 nm excitation at liquid nitrogen temperature and room temperature. The integral emission intensity of the main bands increases by five times, and all of the bands become narrower upon cooling the sample. Similar behavior of emission spectra at low temperatures was observed by Sofich et al.^23^ It is noted that from 300 to 83 K, the peak at 481 nm (20,790 cm^–1^) is increased by a factor of 3.7, and the peak at 604.5 nm (16,542 cm^–1^) is increased by a factor of 4.8. Upon 444 nm excitation of the samples at room temperature, Pr^3+^ ions in the ^3^H_4_ ground state are excited to the ^3^P_2_ level. Then, nonradiative relaxation populated ^3^P_1_, ^3^P_0_, and ^1^D_2_ levels; the emission spectrum consists of nine sets of lines related to the transitions from these levels to the ground state (Figure 5b). These electronic transitions are listed in Table S1 in the Supporting Information. The emission bands at 83 K are split into several components since the f orbitals are shielded by the electric field of the ligand. Table S2 in the Supporting Information provides the energy values of the Stark components and their respective assignments. The results obtained are in good agreement with the results of studies of trivalent praseodymium in other matrices.^24,25^ The excitation line around 588 nm at 83 and 300 K is attributed to the ^3^H_4_ → ^1^D_2_ transition (Figure 5c). Figure 5d shows the emission spectrum associated with the transition from ^1^D_2_ to the ground state, recorded at 83 K. The ^1^D_2_ → ^3^H_4_ transition consists of three well-resolved lines with maxima at 16463 cm^–1^, 16665 cm^–1^, and 16703 cm^–1^. A low-intensity ^1^D_2_ → ^3^H_5_ transition is observed at 14395 cm^–1^. As a result of spin-forbidden transitions from the ^1^D_2_ level to the 3H_J_ level, no radiation was observed at room temperature upon excitation at a wavelength of 588 nm.

**Figure 6:**
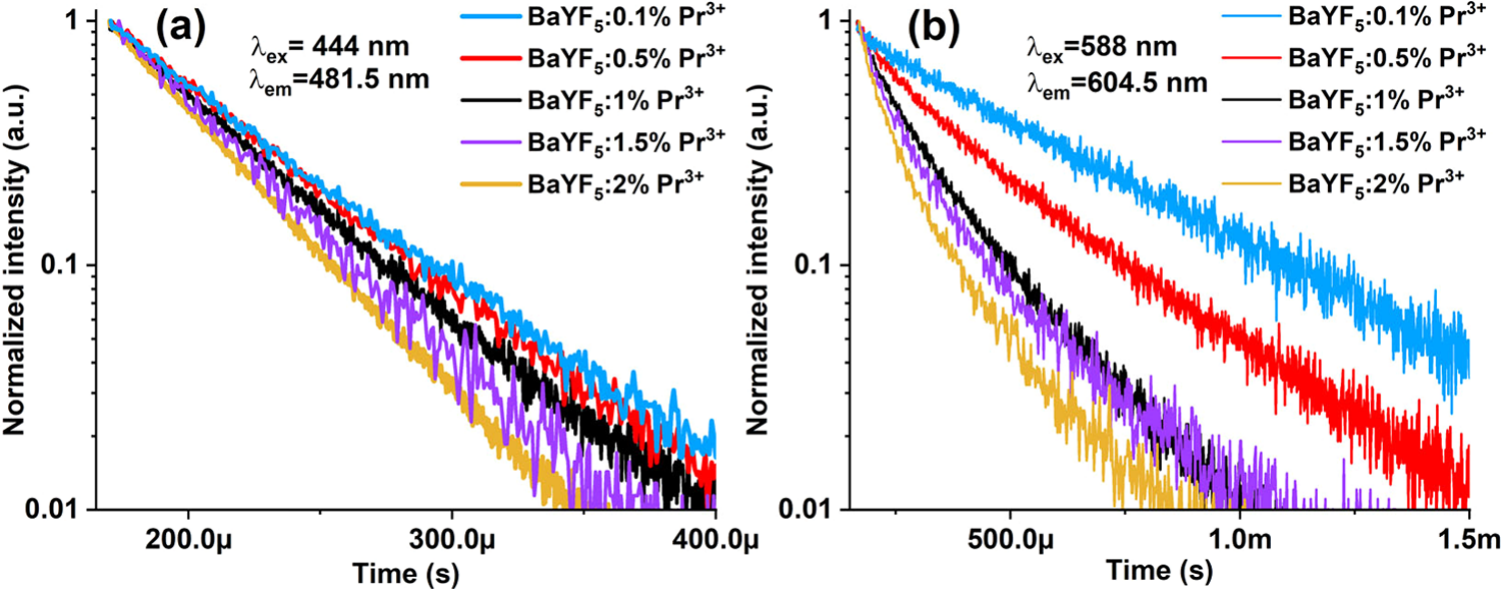
Emission decay curves of BaYF_5_:Pr^3+^ nanoparticles for (a) ^3^P_0_ (λ_ex_ = 444 nm) and (b) ^1^D_2_ (λ_ex_ = 588 nm) Pr^3+^ transitions by monitoring 481.5 and 604.5 nm emission, respectively.

The concentration-dependent emission decay profiles of ^3^P_0_ and ^1^D_2_ transitions for BaYF_5_:Pr^3+^ nanoparticles are exhibited in Figure 6a,b. They are well-fitted using single and double exponential functions, respectively (Table 2). The lifetimes in both cases decrease with increasing concentration of Pr^3+^ ions due to concentration quenching; the same behavior is observed for other matrices.^26,27^

**Table 2:** Emission Lifetime Values for ^3^P_0_ and ^1^D_2_ Transitions of Pr^3+^ in BaYF_5_:Pr^3+^ Nanoparticles

		^1^D_2_	
compounds	^3^P_0_ (μs)	τ_1_ (μs)	τ_2_ (μs)
BaYF_5_:0.1%Pr^3+^	51.2	53.3	446.0
BaYF_5_:0.5%Pr^3+^	49.4	77.3	311.0
BaYF_5_:1%Pr^3+^	45.2	57.2	195.3
BaYF_5_:1.5%Pr^3+^	40.0	40.0	172.4
BaYF_5_:2%Pr^3+^	36.9	35.2	144.2

A strong dependence of cross-relaxation (CR) processes on Pr^3+^ concentration for the ^1^D_2_ level and much weaker for the ^3^P_0_ level was observed. Similar behavior was seen for many hosts doped with praseodymium ions.^24^ An educative example could be found in the paper presenting the dependence of multiphonon nonradiative transition rate *W*_MNR_ on the distance between lanthanide ion levels in the LaAlO_3_ crystal.^28^ Despite the enormous energy gap Δ*E* of about 6500 cm^–1^ between the two adjacent levels, i.e., ^1^D_2_ and ^1^G_4_, the *W*_MNR_ was not subject to the energy gap law, which relates the *W*_MNR_ to the number of phonons bridging Δ*E*^29^ (see Figure 1 in ref (28)). If five or more phonons are needed to bridge this distance, then nonradiative multiphonon transitions should be practically negligible. The authors explained this observation by the presence of the strong CR^24,28^ represented as the [^1^D_2_, ^3^H_4_] → [^1^G_4_, ^3^F_3,4_] transition, which formed another channel depopulating the ^1^D_2_ level. It should be noted that the ^3^P_0_ level was not depopulated by CR as strongly with increasing concentration of Pr^3+^ ions in the matrix as the ^1^D_2_ level, although the [^3^P_0_, ^3^H_4_] → [^1^G_4_, ^1^G_4_] CR is also possible taking into account that here it would also be a process with perfect energy matching.

Cross-relaxation is a process occurring in a pair of lanthanide(III) ions, here Pr1 and Pr2. From the initial state [^3^P_0_, ^3^H_4_] a pair passes to the final state [^1^G_4_, ^1^G_4_] in two simultaneous transitions: ^3^P_0_ → ^1^G_4_ for Pr1 and ^3^H_4_ → ^1^G_4_ for Pr2. Likewise, the pair of Pr^3+^ ions being in the state [^1^D_2_, ^3^H_4_] passes to a more energetically stable state in the two transitions ^1^D_2_ → ^1^G_4_ and ^3^H_4_ → ^3^F_3,4_. Please note that for the Pr1 and Pr2 pair in the [^3^P_0_, ^3^H_4_] state, both CR transitions are spin-forbidden, unlike the transitions for the pair in the state [^1^D_2_, ^3^H_4_] for which they are spin-allowed and therefore more likely. As a result, CR from the ^1^D_2_ level is much more effective.

**Figure 7:**
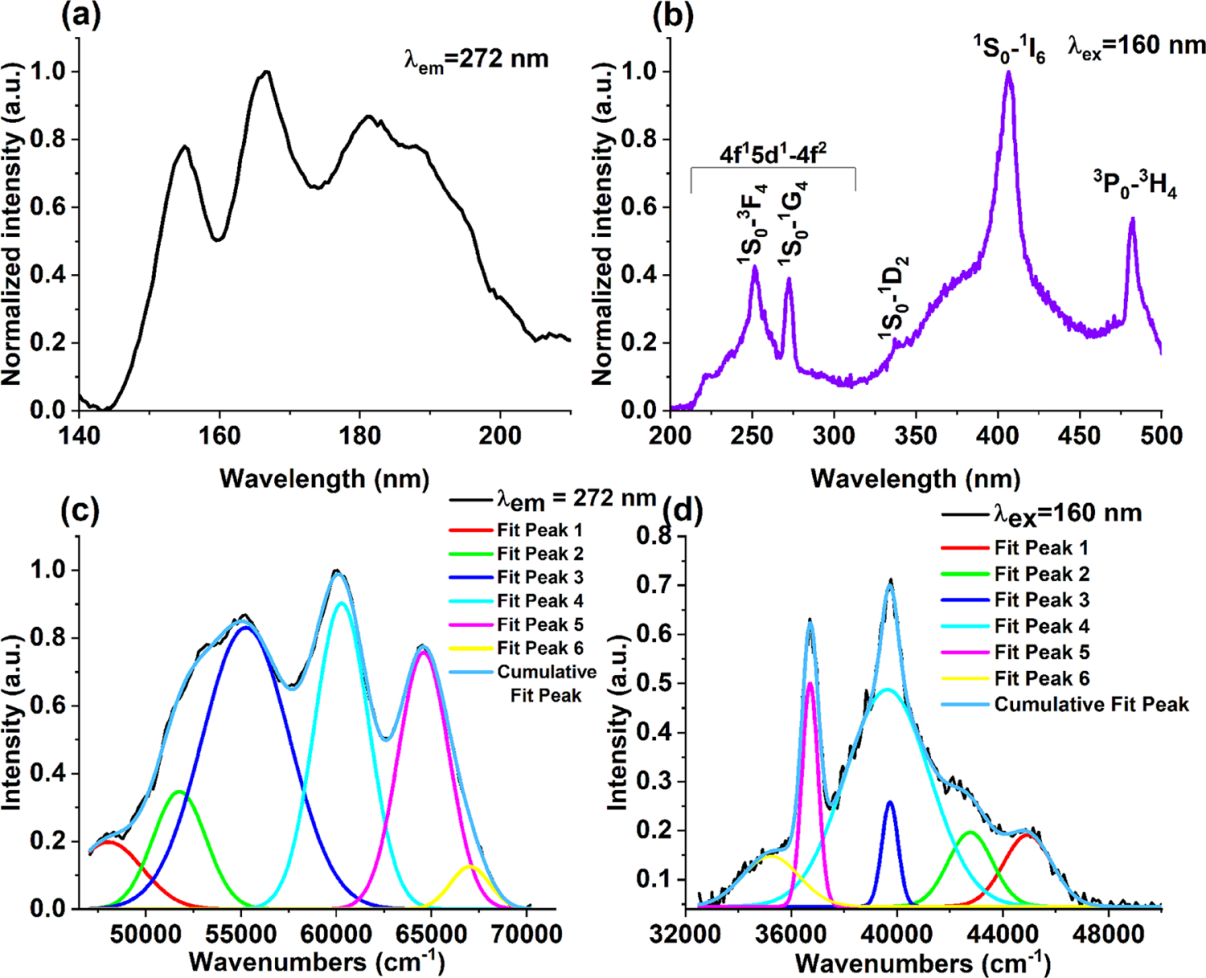
(a) Excitation spectrum monitored at 272 nm emission and (b) emission spectrum at 157 nm excitation in BaYF_5_:1%Pr^3+^ at 300 K. The decomposition of the (c) excitation band and (d) emission band in the ultraviolet region into Gaussian components of BaYF_5_:1%Pr^3+^.

Figure 7a illustrates the excitation spectra of the BaYF_5_:1%Pr^3+^ sample under monitoring the wavelength of 272 nm at room temperature. The excitation spectrum was attributed to the interconfigurational transition from the ground level (4f^2^) of the Pr^3+^ ion to the levels of the 4f5d electronic configuration.^30^ Six main Stark components of the 4f5d electronic configuration were observed with maximum absorption at 48,039, 51,762, 55,254, 60,279, 64,582, and 67,013 cm^–1^ (Figure 7c).

If the lower 4f5d level of Pr^3+^ is located above the ^1^S_0_ state, then upon ultraviolet excitation of the Pr^3+^ ion relaxation occurs from the lower 4f5d level to the ^1^S_0_ level. It is followed by a photon cascade emission: the first photon is generated because of the ^1^S_0_ → ^1^I_6_ emission transition and the second photon is born as a result of the ^3^P*_j_* → ^3^H*_J_* emission. Figure 7b shows the emission spectrum of BaYF_5_:1%Pr^3+^ upon excitation at 160 nm at room temperature. It is obvious that the emission spectrum shows the simultaneous occurrence of inter- and intraconfiguration transitions (4f5d → 4f^2^ and ^1^S_0_ → 4f^2^).^31^ Therefore, one would expect that state ^1^S_0_ is below or very close to the lowest 5d state. In the emission spectrum of BaYF_5_:1%Pr^3+^, narrow luminescent lines at about 251, 272, 336, and 406 nm are observed according to the transitions from the ^1^S_0_ to the ^3^F_4_, ^1^G_4_, ^1^D_2_, and ^1^I_6_ states, respectively. The ^1^S_0_–^3^H*_j_* transition at ∼217 nm is not observed in the luminescence spectrum because it contributes only 1% of the total emitted light.^32^ A likely explanation for the presence of the ^3^P_0_ → ^3^H_4_ transition of the 4f^2^ electrons of the Pr^3+^ ions is the nonradiative relaxation that occurs from the 4f^1^5d^1^ excited state via the crossing point of these two electronic configurations. Another explanation could be related to a cascade of photon emissions, which requires more experiments to prove.^33^

In the range of 210–300 nm, in addition to the sharp emission of lines from the ^1^S_0_ state, the 4f5d–4f^2^ emission transition is observed. The broad band between 300 and 500 nm is likely the result of self-trapped excitons that are created by irradiating a sample with energy greater than the band gap. Similar emission was observed and studied in detail in NaYF_4_:Pr^3+^.^34^

**Figure 8:**
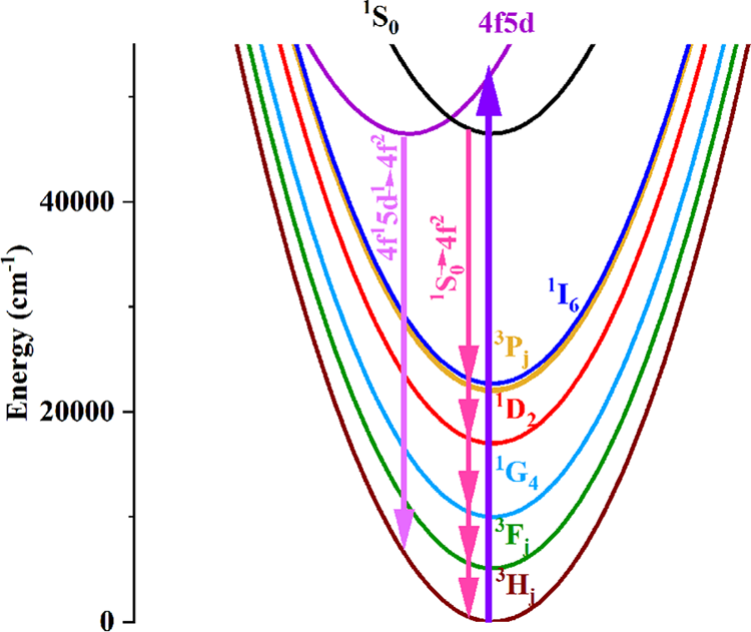
Schematic single configurational coordinate diagram for BaYF_5_:Pr^3+^ nanoparticles. Some 4f^2^ levels have been omitted in this figure for clarity.

The emission spectra in the ultraviolet region were deconvoluted into six Gaussian components: 44,939, 42,767, 39,731, 39,634, 36,709 and 35,231 cm^–1^ (Figure 7d). The Stokes shift (Δ*S*) and the lowest energy level 4f^1^5d^1^ (*E*_fd_) were estimated using the equations
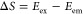
3
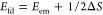
4where *E*_ex_ is the position of the lowest energy absorption maximum and *E*_em_ is the position of the highest energy emission maximum. According to eqs 3 and 4, the Stokes shift is 3100 cm^–1^, and the lowest energy level 4f^1^5d^1^ is 46,489 cm^–1^. Thus, the position of the 4f^1^5d^1^ and ^1^S_0_ levels are very close, since the position of the ^1^S_0_ level is practically independent of the influence of the crystal field and is equal to approximately 46,500 cm^–1^.^35^Figure 8 shows the schematic single configuration coordinate diagram of BaYF_5_:Pr^3+^ nanoparticles. In this figure, for the sake of clarity, some of the 4f^2^ energy levels have been omitted.

**Figure 9:**
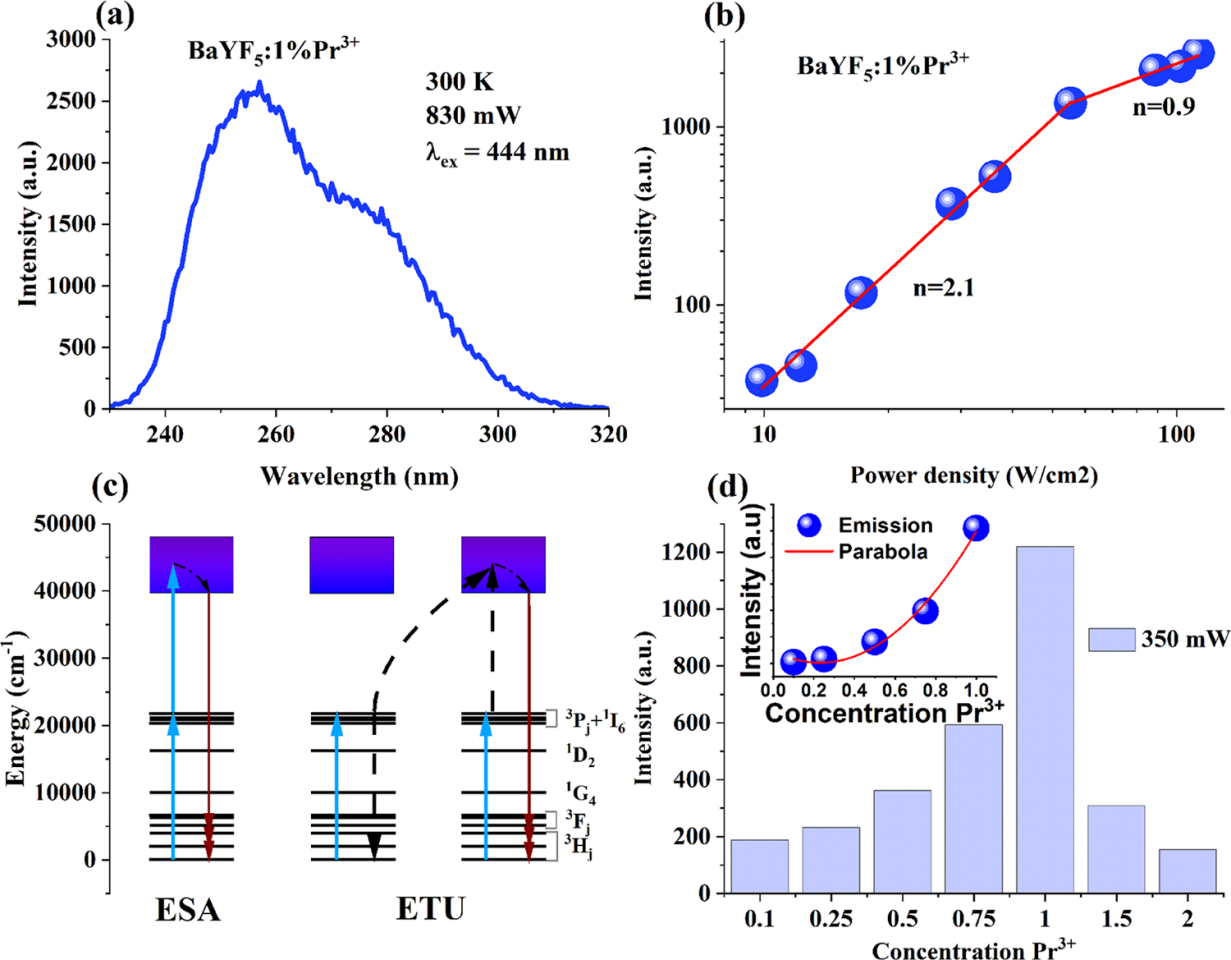
(a) Upconversion spectrum of BaYF_5_:1%Pr^3+^ nanoparticles upon 444 nm laser excitation. (b) Dependence of the upconversion intensity on pump power for BaYF_5_:1%Pr^3+^. (c) Schematic energy-level diagram and the possible upconversion mechanism. Solid arrows represent absorption and emission processes, while dashed arrows indicate nonradiative transitions. (d) Upconversion intensity depends on Pr^3+^ doping concentration. (inset) The intensity of upconversion luminescence data was modeled using a parabolic function.

Figure 9a presents the upconversion spectrum of the BaYF_5_ sample doped with 1% Pr^3+^ upon excitation with a 444 nm laser (830 mW). The emission spectrum was observed between 250 and 315 nm, with the maximum emission intensity at ∼257 nm. This band can correspond to the 4f^2^ → 4f^1^5d^1^ transition of Pr^3+^.

It is known that the intensity of upconversion, denoted as *I*, is proportional to the nth power of the pump power (*P*)^36^
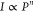
5where *n* is the number of photons required to produce one upconversion photon. The value of *n* can be determined from the slope of the linear plot of log *I* versus log *P*. The relationship between the upconversion luminescence intensity at 257 nm for the BaYF_5_:1%Pr^3+^ sample and the pump power of the 444 nm laser is illustrated in Figure 9b. For the excitation power density up to 36.3 W·cm^–2^, the calculated slope value is 2.146 ± 0.080 (*R*^2^ = 0,99443), which indicates that two photons are involved in the upconversion process. However, for power density higher than 36.3 W·cm^–2^, the slope changes to 0,864 ± 0,087 (*R*^2^ = 0,98023), indicating the occurrence of the saturation effect. This effect is a consequence of the competition between upconversion and linear decay of the population of the metastable excited state (here is the ^3^P_0_ level) due to radiative and nonradiative transitions.^37^ Two known processes are responsible for two-photon upconversion: excited state absorption (ESA) and energy-transfer upconversion (ETU).^38^Figure 9c shows the schematic energy-level diagram of the Pr^3+^ ion excited by a 444 nm laser and the potential upconversion mechanisms. Only one Pr^3+^ ion is involved in the ESA mechanism. Pr^3+^ in the ground state absorbs a single photon and transitions into an intermediate excited state (^3^P_2_). After sequentially absorbing a second photon, it can enter the excited state (4f5d) and emit higher-energy photons (UV-C light) upon returning to the ground state (Figure 9c). The ETU upconversion mechanism involves a pair of neighboring Pr^3+^ ions, with one of them serving as the sensitizer and the other as the activator. A pair of adjacent Pr^3+^ ions is excited from the ground state to the ^3^P_2_ state by two photons (444 nm). One of the excited ions transfers its excitation energy to a neighboring excited ion and subsequently returns nonradiatively to the ground state. Upon receiving additional energy, the second excited ion is elevated to the 4f5d state. Finally, the Pr^3+^ ion from the 4f5d state radiatively relaxes to the ground state and produces ultraviolet radiation.

The distance between the sensitizer and activator ions affects the efficiency of the ETU upconversion mechanism. Therefore, to establish the optimal trivalent praseodymium concentration, the dependence of the upconversion intensity on the activator concentration was explored. Figure 9d shows the upconversion emission intensity of BaYF_5_:1%Pr^3+^ samples upon the same excitation (444 nm, 350 mW). As the concentration of Pr^3+^ in BaYF_5_ increases, the upconversion intensity increases, reaching its peak at 1%. However, the effect of concentration quenching leads to a significant decrease in the intensity of upconversion with a further increase in the Pr^3+^ concentration. For our samples with the Pr^3+^ concentration “*X*” ranging from 0.1 to 1%, the correlation between the upconversion intensity “*I*” and the Pr^3+^ concentration could be fitted by a quadratic function, namely, *I* ∝ *X*^2^ (see inset in Figure 9d). According to Tanner, the upconversion luminescence intensity resulting from ETU changes quadratically.^39^ This suggests that the mechanism responsible for upconversion in our study is most likely ETU. While we cannot discount the possibility that ESA may also contribute to upconversion, our next article will scrutinize the upconversion mechanism in greater detail.

It should be noted that despite the small size of nanoparticles, the BaYF_5_:Pr^3+^ phosphor exhibits strong UV emission when exposed to VUV and a 444 nm laser. The unique properties of this material can be used for various applications, such as phosphor-based lighting systems, UV-sensitive detectors, or luminescent markers for biological research.

## Conclusions

BaY_1–*x*_Pr_*x*_F_5_ (0 ≤ *x* ≤ 0.01) nanoparticles with an average size of 26–37 nm were synthesized by the microwave hydrothermal method. The intraconfigurational 4f–4f transitions of Pr^3+^ in BaYF_5_ at room temperature and liquid nitrogen temperature have been studied. The intensity of excitation and emission of photoluminescence decreased with increasing temperature. The fluorescence decay curves of ^3^P_0_ and ^1^D_2_ level emission have been studied. The ^1^D_2_ energy level is more sensitive to concentration quenching than the ^3^P_0_ level. The former one is effectively depopulated by spin-allowed CR transitions. The interconfigurational 4f5d–4f transition of Pr^3+^ at room temperature has been investigated. Upconverted ultraviolet emission in the range of 230 to 315 nm is observed in BaYF_5_:Pr^3+^ when the samples undergo 444 nm laser excitation. Two-photon absorption is responsible for upconversion luminescence in UV-C. The sample with 1% Pr^3+^ has the highest upconversion emission. The Pr^3+^-doped BaYF_5_ nanoparticles are a promising candidate for visible and UV-C phosphors, which open up new possibilities for UV-requiring applications, such as security industries, phototherapy, and sterilization.

## Data Availability

The data presented in this study will be made available on Zenodo with the DOI: 10.5281/zenodo.10050916 upon acceptance by the journal.

## Supporting Information Available

Tables of electronic transitions and Stark levels of the Pr^3+^ ion in BaYF_5_ (PDF)

## Notes

For the purpose of open access, the authors have applied a CC-BY public copyright license to any Author Accepted Manuscript (AAM) version arising from this submission.
