# Supplementary material for: Upconversion Luminescence Properties of Pr3+-Doped BaYF5 Nanoparticles Prepared by Microwave Hydrothermal Method
Source: Inorg Chem. 2024 Jan 31;63(6):3028–36. doi: 10.1021/acs.inorgchem.3c03821 (PMC10865355; doi:10.1021/acs.inorgchem.3c03821)
Supplement: Supplementary file 1 — ic3c03821_si_001.pdf [file ic3c03821_si_001.pdf]

Supporting Information

Upconversion luminescence properties of Pr^{3+} doped $BaYF_5$ nanoparticles prepared by microwave hydrothermal method

Nadiia Rebrova, Patrycja Zdeb, Karol Lemański, Bogusław Macalik, Oleksii Bezkrovnyi, Przemysław J. Dereń*

Institute of Low Temperature and Structure Research, Polish Academy of Science, ul. Okólna 2, 50-422 Wrocław, Poland.

* Corresponding author e-mail: n.rebrova@intibs.pl

Table S1. Electronic transitions of the Pr^{3+} ion in the $BaYF_5$:1%Pr^{3+} nanoparticles.

Transition	λ (nm)	E (cm^{-1})
$^3P_0 \rightarrow ^3H_4$	481.4	20773

$^3P_0 \rightarrow ^3H_5$	539	18553
$^3P_0 \rightarrow ^3H_6$	604.5	16542
$^3P_0 \rightarrow ^3F_2$	639.5	15637
$^3P_0 \rightarrow ^3F_3$	693.9	14411
$^3P_0 \rightarrow ^3F_4$	717.5	13937
$^3P_1 \rightarrow ^3H_5$	522	19157
$^1D_2 \rightarrow ^3H_4$	608	16447
$^3P_1 \rightarrow ^3F_3$	694	14409

Table S2. The Stark components of the manifolds of Pr^{3+} ions in $BaYF_5$ determined from the emission and excitation spectra.

(S, L, J) multiplet	Experimental Stark levels (cm^{-1})
$^3H_4$	8, 63, 97, 271, 405
$^3H_5$	2153, 2674, 2229, 2300, 2377, 2442
$^3H_6$	4189, 4236, 4269, 4323, 4432, 4507, 4690
$^3F_2$	5085, 5098, 5132, 5168, 5398
$^3F_3$	6475, 6547
$^3F_4$	6872, 6893, 6919, 6955, 7020
$^1G_4$	9791

$^1D_2$	16880, 17000, 17141
$^3P_0$	20776
$^3P_1$	21423, 21320
$^1I_6$	21566, 22146
$^3P_2$	22707, 22592, 22476, 22420
